# Lineage-Specific Functionality of an Interferon Regulatory Factor 5 Lupus Risk Haplotype: Lack of B Cell Intrinsic Effects

**DOI:** 10.3389/fimmu.2018.00996

**Published:** 2018-05-07

**Authors:** Justine Calise, Susana Marquez Renteria, Peter K. Gregersen, Betty Diamond

**Affiliations:** ^1^PhD Program in Molecular Medicine, Donald and Barbara Zucker School of Medicine at Hofstra-Northwell, Hempstead, NY, United States; ^2^Laboratory of Autoimmune & Musculoskeletal Diseases, The Feinstein Institute for Medical Research, Center for Autoimmune, Musculoskeletal, and Hematopoietic Diseases, Northwell Health, Manhasset, NY, United States; ^3^Laboratory of Genomics & Human Genetics, The Feinstein Institute for Medical Research, Center for Genomics and Human Genetics, Northwell Health, Manhasset, NY, United States

**Keywords:** genetics, B lymphocytes, monocytes, haplotypes, autoimmunity, lupus

## Abstract

Interferon regulatory factor 5 (IRF5) is widely recognized as a risk locus for systemic lupus erythematosus (SLE). Risk gene and IRF5 activation is triggered through toll-like receptor signaling. In myeloid cells, this leads to production of type I interferon and inflammatory cytokines, with enhanced production in cells of individuals harboring IRF5 risk alleles. Mouse models have also demonstrated the importance of IRF5 in B cell function, particularly plasma cell differentiation and isotype switching. Here, we evaluated the major SLE risk haplotype of IRF5 on the functional attributes of freshly isolated B cells from human subjects who do not have evidence of SLE or other forms of autoimmunity. We took this approach to avoid the complications of studying genotype-phenotype relationships in B cells that have been chronically exposed to an inflammatory disease environment before isolation. We focused on B cell endophenotypes that included gene expression, antibody secretion, class switching, and apoptotic susceptibility. We performed IRF5 overexpression studies, genetic reporter assays and electro-mobility shift assays on B and myeloid cell lines. Somewhat surprisingly, the results of our analyses indicate that IRF5 risk genotypes do not have a B cell intrinsic effect on these B cell functions. By contrast, we confirmed that the IRF5 risk and non-risk haplotypes exert differential effects in myeloid cells, including an increased susceptibility to apoptosis conferred by the risk haplotype. We also demonstrated an increased binding of the transcription factor specificity protein 1 to an insertion/deletion present in the risk haplotype. Our findings raise the specter that genetic risk alleles can have complex and unexpected lineage-specific effects, and these must be carefully considered when guiding or developing therapies based on understanding disease risk haplotypes.

## Introduction

Systemic lupus erythematosus (SLE) is an autoimmune disease characterized by a breach in B cell tolerance to self-antigens. Genetic, hormonal, and environmental factors contribute to susceptibility to SLE. The transcription factor interferon regulatory factor 5 (IRF5) is one of approximately 100 genes with SLE-associated risk variants (1, 2). Within the immune system, IRF5 is expressed in myeloid cells and lymphocytes (3–5). IRF5 has diverse roles in myeloid cells including production of interferon-α (IFNα) and other proinflammatory cytokines (6), regulation of the cell cycle and apoptosis (7), macrophage polarization (3), and metabolism (8). IRF5 can be activated by toll-like receptor (TLR) agonists, including nucleic acid-containing immune complexes present in SLE. IFNα can contribute to B cell autoreactivity seen in SLE. Many SLE patients have elevated levels of serum IFNα and markedly increased expression of interferon-inducible genes (9–12). Prior studies have shown that SLE-associated risk alleles of IRF5 display increased expression in myeloid cells and influence monocyte and macrophage activation (8, 13, 14).

IRF5 has direct effects on B cells as well. IRF5 has been shown to be critical for terminal B cell differentiation to plasma cells in mice (15, 16). IRF5 also plays a role in isotype switching to IgG. *Irf5^−/−^* mice have increased levels of IgG1 and decreased levels of IgG2c (17). IRF5 has been shown to directly regulate transcription of the γ2a locus; *Irf5^−/−^* mice do not produce IgG2a antibodies (18). There is evidence that IRF5 is necessary for SLE development based on studies of pristane-treated C57BL6 *Irf5^−/−^* and MRL/lpr *Irf5^−/−^*mice. The former lack antinuclear antibody (ANA) titers and glomerular deposits of immune complexes after pristane challenge (18). The latter survive longer, exhibit milder glomerulonephritis and lower ANA titers (16, 19) than IRF5 sufficient MRL/lpr mice. Consistent with a contribution of IRF5 to autoimmunity, and a contribution of lupus-like inflammation to IRF5 expression, the autoimmune C57BL/6.*Nba2*, NZB/W, and *Sle123* mouse strains all exhibit increased expression of IRF5 in splenic cells compared with C57BL/6 mice (20).

FcγRIIb is known to protect against autoantibody production (21, 22). When bound to IgG immune complexes and co-ligated to the BCR, FcγRIIb initiates an inhibitory signaling cascade, mediated through its immunoreceptor tyrosine-based inhibitory (ITIM) motif (22, 23). In mice, a reciprocal regulation of IRF5 and FcγRIIb has been reported (20). FcγRIIb is important for B cell tolerance by setting a cellular activation threshold. C57BL6.*Nba2* mice develop a lupus-like phenotype due to the presence of the *Nba2* locus (24). C57BL6.*Nba2 Irf5^−/−^* mice exhibit increased expression of FcγRIIb and C57BL6 *Fcgr2b^−/−^* mice exhibit increased expression of IRF5 (20), suggesting reciprocal regulation of IRF5 and FcγRIIb.

Located on chromosome 7 in humans, IRF5 has a total of 12 exons. Exons 2–8 and part of 9 are coding. Exon 1 is subdivided into four non-coding exons 1a–1d (25). Each non-coding exon corresponds to a different promoter (26), allowing alternative splicing of the gene. There are over 100 known polymorphisms of IRF5, but only four are thought to be functional (27). Three of these polymorphisms are located in non-coding regions of IRF5. The non-coding polymorphisms rs142738614, rs2004640, and rs10954213, are located between exons 1d and 1a, in exon 1b, and in the polyA tail of exon 9, respectively. The three alleles have been reported to be in linkage disequilibrium (LD) (13). The fourth polymorphism is a 30 bp insertion/deletion (indel) located in exon 6, and inherited independently of the three SNPs.

The “T” risk allele of SNP rs2004640 is located in exon 1b and introduces a donor RNA splice site, enabling expression of mRNAs containing exon 1b (2). Exon 1b transcripts are not translated into protein (28) and are expressed at very low levels compared with exon 1a transcripts (29), so the functional significance of rs2004640 is not entirely clear. The “A” risk allele of the SNP rs10954213 in the 3′ UTR of exon 9 introduces a more proximal polyA site. This allele has been shown to confer increased expression as well as greater mRNA stability likely due to decreased susceptibility to degradation of the shorter transcripts (30, 31). The polymorphism rs142738614 is an indel located 64 bp upstream of exon 1a that refers to the number of copies of the 5 bp sequence CGGGG; the risk allele has four copies which introduces an additional binding site for the transcription factor specificity protein 1 (SP1) (26, 32). To date, the functional impact of this additional SP1 binding site in predisposition to SLE is unknown.

Currently, data available on the effects of IRF5 risk alleles in human B cells are rather limited. In contrast to previous reports, using cell lines or B cells of SLE patients (2, 33), we demonstrate that in healthy donors, the IRF5 risk and non-risk haplotypes are not differentially expressed in B cells in the resting state or after TLR activation. In addition, IRF5 haplotypes do not differentially regulate B cell differentiation to antibody-secreting cells (ASCs) or IRF5-mediated apoptosis, and that IRF5 does not regulate FcγRIIb expression in human B cells. Our findings confirm that these same IRF5 risk haplotypes do exert differential effects in myeloid cells and demonstrate that the 4× CGGGG indel is a potential causal allele in this context. Thus, we conclude that IRF5 acts indirectly on B cells through B cell extrinsic pathways. These data underscore the importance of examining multiple cell types when studying risk haplotypes in complex diseases. Moreover, they clearly demonstrate that the observation that a gene is important in the function of a particular cell type (e.g., the importance of IR5 in B cell function) does not necessarily imply that disease-associated haplotypes will display differential effects intrinsic to that cell type.

## Materials and Methods

### Study Subjects and Study Approval

Study participants came from the Genotype and Phenotype Registry (GAP) registry at the Feinstein Institute. Their ancestry was European (EUR), African (AFR), Hispanic/Latino, South Asian (SAS), West Asian, or any combination of these. Subjects included premenopausal females and age-matched males, from 18 to 50 years of age. All subjects included in the study were homozygous risk or homozygous non-risk for IRF5, without autoimmune disease and not on corticosteroids, chemotherapeutic or cytotoxic drugs, or selective cell depletion therapies, thereby allowing us to study effects of the risk haplotype independent of the effects of disease or medications. The study was approved by the Institutional Review Board at the Feinstein Institute for Medical Research. Prior written consent was received from all study participants.

### IRF5 Haplotype Assembly

Phase 3 genetic data for the IRF5 locus on chromosome 7 were extracted from the 1000 Genomes Project browser^1^ for the following populations: AFR, African; AMR, American/Hispanic/Latino; EAS, East Asian; EUR, European; SAS, South Asian. Haplotype maps were constructed for each population using Haploview software. Genotyping for representative variants (see Genotyping) was performed on subjects in the GAP.^2^

### Genotyping

We confirmed the LD of rs142738614, rs2004640, and rs10954213 by selecting subjects from the GAP Registry that had been genotyped for rs2004640 and rs10954213 on the ImmunoChip (Illumina). The subjects we selected to further genotype for rs142738614 were homozygous for rs10954213. Genotyping for rs2004640 and rs10954213 was performed on the ImmunoChip by the Center for Genomics and Human Genetics at the Feinstein Institute. For rs142738614 genotyping, genomic DNA was obtained from 700 subjects homozygous for rs10954213 with membership in the GAP registry. The genotyping PCR was performed using the AmpliTaq Gold^®^ DNA Polymerase with Buffer II and MgCl_2_ kit (ThermoFisher Scientific), dNTPs (Takara), DMSO (Sigma), and PCR-grade H_2_O. Primers used for genotyping were as follows: forward 5′ CTGCAGTTGCCAGGTCAGT 3′, reverse 5′ CGGACGCAGAGAGGAGAG 3′. Final concentrations of Taq, dNTPS, DMSO, MgCl_2_, primers, and DNA were 0.05 U/μl, 0.2 mM, 5%, 1 mM, 0.1 µM, and 0.8 ng/µl, respectively. The touchdown PCR utilized the following cycling parameters: 95°C for 10 min, 12 cycles of: 94°C for 30 s, 62°C for 30 s (0.5°C decrease/cycle), 72°C for 1 min, followed by 30 cycles of 94°C for 30 s, 56°C for 30 s, 72°C for 1 min, followed by 1 cycle of 72°C for 5 min. After the PCR, products were subjected to treatment with ExoSAP-IT^®^ (Affymetrix) according to the manufacturer’s instructions. PCR products were then sent to GENEWIZ (South Plainfield, NJ, USA) for sequencing.

### Human Peripheral Blood Peripheral Blood Mononuclear Cell (PBMC) and B Cell Isolation

Up to 50 ml of peripheral blood was drawn from consenting subjects in the GAP Registry at the Feinstein Institute for Medical Research. Blood was collected in heparinized tubes and diluted 1:1 with Hank’s Balanced Salt Solution (HBSS). Diluted blood was overlaid onto Ficoll-Paque PLUS density gradient media (GE Healthcare) and centrifuged at 400 × *g* for 30 min at 25°C with the brake and acceleration turned off. The buffy coat layer was isolated, washed with HBSS, and resuspended in staining buffer [HBSS + 2% heat inactivated fetal bovine serum (FBS) + 1 mM EDTA]. PBMCs were counted and used for further experiments or when necessary, B cell isolation was performed with the EasySep Human B Cell Enrichment Kit (Stemcell Technologies) or the EasySep Human Naïve B Cell Enrichment Kit according to the manufacturer’s instructions.

### Cell Lines and Primary Cell Culture

Ramos (ATCC^®^ CRL-1596™), Raji (ATCC^®^ CCL-86™), Daudi (ATCC^®^ CCL-213™), Jurkat (ATCC^®^ TIB-152™), and THP-1 (ATCC^®^ TIB-202™) cells were obtained through the American Type Culture Collection and cultured according to ATCC recommendations in RPMI supplemented with 10% FBS and 1% penicillin/streptomycin (ThermoFisher Scientific). Primary B cells and PBMCs were cultured in RPMI supplemented with 10% FBS and 1% penicillin/streptomycin.

### Treatment of B Cells With IRF5-Activating Agents

Primary B cells were seeded into 96-well plates in culture medium with final concentrations of 2.5 µM CpG ODN 2006 (Invivogen), 5 µg/ml R848 (Resiquimod, Invivogen), or medium alone. Cells were incubated at 37°C in a humidified 5% CO_2_ incubator until further analysis.

### RNA Isolation and cDNA Synthesis

Freshly sorted or treated B cells were pelleted and stored in Trizol at −80°C until RNA isolation. RNA isolation was performed with the Direct-Zol RNA Miniprep or Microprep Kit (Zymo Research) according to the manufacturer’s instructions. Freshly isolated RNA was reverse transcribed into cDNA using the iScript cDNA Synthesis Kit (BioRad) according to the manufacturer’s instructions and stored at −20°C.

### Quantitative Real-Time PCR

All TaqMan assays were obtained from ThermoFisher Scientific. In all experiments, mRNA levels were normalized to levels of the endogenous control *HPRT1* (Hs99999909_m1). TaqMan assays used included *IRF5* (Hs00158114_m1), *FCGR2B* (Hs01634996_s1), *SP1* (Hs00916521_m1), and *CD86* (Hs01567026_m1). For IRF5 expression in sorted B cell subsets and monocytes, cDNA was first subjected to preamplification for *HPRT1* and *IRF5* using the TaqMan^®^ PreAmp Master Mix Kit (ThermoFisher Scientific) according to the manufacturer’s instructions. All qPCR reactions were set up in 384-well format with master mix consisting of LightCycler 480 Probes Master (Roche), TaqMan assay, and sterile PCR-grade H_2_O, with 1 µl cDNA per reaction well (5 µl cDNA for PreAmp samples). Reactions were performed in duplicate. All TaqMan assays were done on a Roche LightCycler 480 using the following cycling parameters: one cycle of 95°C for 10 min, and 45 cycles of 95°C for 20 s followed by 60°C for 40 s. Analysis of sterile transcripts was done using LightCycler 480 SYBR Green I Master (Roche), sterile PCR-grade H_2_O, and 1 µl cDNA per reaction well, in duplicate. SYBR Green assays were done on a Roche LightCycler 480 using the following cycling parameters as reported previously (34): one cycle of 95°C for 10 min, and 40 cycles of 94°C for 1 min, 58°C for 1 min, and 72°C for 1 min. A melting curve analysis was also performed with continuous acquisition starting at 97°C and ending at 40°C. Primer sets for sterile transcripts included *HPRT1*: forward 5′ TGCAGACTTTGCTTTCCTTGGTCAGG 3, reverse 5′ CCAACACTTCGTGGGGTCCTTTTCA 3′, and germline IgG: forward 5′ TCCTCTCAGCCAGGACCAA 3, reverse 5′ TCTTGGCATTATGCACCTCC 3′ (34). The final concentration of primers used was 0.1 µM. For baseline expression experiments, relative mRNA expression levels were calculated using the ΔCt method, 2^(Ct endogenous control − Ct gene of interest)^. For experiments involving treatment of B cells with IRF5 activating agents, relative mRNA expression levels were calculated using the ΔΔCt method, 2^((Ct gene of interest, untreated − Ct endogenous control, untreated) − (Ct gene of interest, treated − Ct endogenous control, treated))^.

### Fluorescence-Activated Cell Sorting

For B cell subset sorting, cryopreserved PBMCs were thawed and stained with the following monoclonal antibodies for 30 min on ice in the dark: αIgD-FITC, αCD27-PE, αCD38-PE-Texas Red (BD Biosciences), αCD10-PE-Cy7, αCD19-APC (BioLegend), and αCD14-Pacific Blue (BD Biosciences). For sorting of transfected Raji and Daudi B cells, cells were transfected with either empty or IRF5 overexpressing vectors with a cyan fluorescent protein (CFP) overexpressing reporter vector (see Transient Transfection and IRF5 Overexpression), and 24 h later prepared for sorting by pelleting and resuspended in staining buffer. Immediately before sorting, 1 µl propidium iodide (PI) (ThermoFisher Scientific) was added to the cells. PI negative, CFP-positive cells were sorted, pelleted, and stored in Trizol at −80°C. In all experiments, cells were sorted using a BD FACSAria or BD FACSAria SORP.

### Flow Cytometry

The monoclonal antibody 2B6 that was used for FcγRIIb staining has been described previously (35). All cell surface staining conditions took place in staining buffer for 30 min on ice in the dark. For baseline FcγRIIb expression in B cell subsets, cryopreserved PBMCs from genotyped GAP subjects were thawed and incubated with the following monoclonal antibody cocktail: αFcγRIIb-AF488 (Macrogenics), αIgD-PE, αCD27-APC, αCD19-PerCP, αCD38-APC-Cy7, αCD10-PE-Cy7 (BioLegend), αCD14-Pacific Blue (BD Biosciences), and as a dump gate αCD3 and αCD16, both AF700 conjugated. The fixable viability dye eFluor506 was included for live/dead discrimination (eBioscience). For analysis of stimulated primary B cells, cells were incubated with eFluor660 fixable viability dye, αCD19-BV421 (BioLegend) and αFcγRIIb-AF488. For measurement of FcγRIIb in IRF5 overexpression experiments, cells were incubated with the fixable viability dye eFluor660 and αFcγRIIb-AF488. Only CFP^+^ cells were considered in the analysis. For analysis of antibody-secreting cultured B cells, on the fourth day of culture B cells were stained with αCD19-APC-Cy7, αCD27-PE, αCD38-PE-Texas Red, αCD86-BV711 (BioLegend), αCD138-BV421 (BioLegend), αIgM-PerCP-Cy5.5 (BioLegend), and αIgG-PeCy7 (BD Biosciences), and viability dye eFluor506. All experimental data were acquired on a BD LSRII or LSR Fortessa and analyzed using FlowJo software (Treestar).

### Apoptosis Assay

Peripheral blood mononuclear cells or were cultured medium with either vehicle (DMSO) or CPT-11 (Sigma) at concentrations of 50 µM. Cells were incubated for 8 h in a 37°C humidified 5% CO_2_ incubator. After incubation, cells were stained with eFluor506 viability dye, αCD19-APC (BioLegend), and αCD14-Pacific Blue. Cells were then prepared for analysis using the PE anti-active caspase 3 apoptosis kit (BD Biosciences) according to the manufacturer’s instructions. Cells were analyzed on a BD LSR Fortessa.

### ELISpot Assay

On the fourth day of primary B cell culture, Immunolon^®^ flat-bottom 96-well microtiter plates (ThermoFisher Scientific) were coated with goat antihuman IgM (Southern Biotech) at a final concentration of 10 µg/ml in HBSS for 1 h at 37°C. Plates were then washed and blocked with culture medium for 1 h at room temperature. Live cells were counted *via* trypan blue exclusion and seeded as serial 1:2 dilutions in fresh culture medium in the plate in duplicate. Plates were briefly spun to settle cells to the bottom and incubated overnight in a 37°C humidified 5% CO_2_ incubator. Plates were subsequently washed and biotinylated goat antihuman IgM was added at a final concentration of 1.6 µg/ml. The plates were incubated for 2 h at 37°C. Plates were washed again and alkaline phosphatase (AP)-conjugated streptavidin was added at a dilution of 1:1,000 and incubated for 1 h at 37°C. Plates were washed and developing solution was added: 1 mg/ml BCIP (Sigma) in AMP buffer. Color development took place at room temperature in the dark until spots appeared; plates were then rinsed with dH_2_O, dried and counted under a light microscope.

### ELISA

The protocol followed for the ELISA was the same as the ELISpot assay with a few modifications. Costar^®^ 96-well flat-bottom plates were used (Corning) and blocking was done with 3% FBS. IgM standards and sample supernatants were also diluted in 3% FBS and incubated for 1.5 h. The incubation with the secondary antibody, AP-goat anti human IgM (Southern Biotech) was done at a dilution of 1:1,000 and lasted for 1 h. The developing solution consisted of 0.05 M Na_2_CO_3_, 0.001 M MgCl_2_, and phosphatase substrate (Sigma). Absorbance was measured at 405 nm.

### Generation of IRF5- and CPF-Overexpressing Constructs

We obtained a plasmid expressing both IRF5 (NM_001098629.1) and CFP (GenBank: KT878729.1) as a fusion protein (Genecopoeia). Both the IRF5 and CFP ORFs were cloned into the pmaxCloning™ vector (Lonza) separately. For the cloning of CFP, a PCR was done on the donor fusion protein plasmid to introduce HindIII and XhoI sites for more straightforward cloning. The PCR primers used were as follows: forward 5′ GATAAGCTTTCTTGTACA-AAGTGGTTCG 3′, reverse 5′ CACACTCGAGGTAAAAGGACAGG 3′. The PCR consisted of 50 ng plasmid DNA, reaction buffer, *pfuUltra* High-Fidelity DNA polymerase, dNTP mix, 0.1 µM primers, and PCR-grade H_2_O. The reaction buffer, polymerase, and dNTP mix were taken from the QuikChange II Site-Directed Mutagensis Kit (Agilent Technologies). Cycling parameters were as follows: 95°C 30 s, 25 cycles of 95°C for 30 s, 55°C for 1 min, and 68°C for 1 min, followed by one cycle of 72°C for 5 min. The product was purified and restriction digested with XhoI and HindIII. The pmaxCloning™ vector was also digested with XhoI and HindIII and dephosphorylated with Antarctic AP (New England Biolabs). Products were purified and ligated with T4 DNA ligase (New England Biolabs). DH5α-TI^R^ *E. coli* (ThermoFisher Scientific) were transformed and subject to kanamycin resistance selection. Resistant colonies were picked and screened *via* colony PCR using the same primers and cycling conditions of: 95°C for 10 min, 30 cycles of 95°C for 20 s, 56°C for 30 s, 72°C for 1 min, and one cycle of 72°C for 5 min. The PCR mix consisted of the same reagents at the same concentrations as indicated in Section “Genotyping.” PCR products were visualized on a 1% agarose gel with SYBR Safe DNA Gel Stain (ThermoFisher Scientific). A positive colony was further grown in *E. coli* and plasmid was isolated using the ZymoPURE™ Plasmid Maxiprep Kit (Zymo Research). Confirmation of plasmid sequence was done at Genewiz (South Plainfield, NJ, USA) with the forward and reverse primers. For the cloning of IRF5, a PCR was also done on the donor fusion protein plasmid to introduce EcoRI and XhoI sites for more straightforward cloning. The primers used were as follows: forward 5′ TATAGAATTCCCAAG-CTGGCTAGTTAAG 3′, reverse 5′ TATACTCGAGATCGAACCACTTTGTACAAGAAA 3′. The PCR consisted of 150 ng plasmid DNA, PrimeSTAR^®^ HS DNA Polymerase with reaction buffer (Takara), 0.25 µM primers, 0.2 mM dNTPS (Takara), and sterile PCR-grade H_2_O. Cycling conditions were 30 cycles of 98°C for 10 s, 55°C for 15 s, and 72°C for 1 min. The product was purified and restriction digested with XhoI and EcoRI. The pmaxCloning™ vector was also digested with XhoI and EcoRI. Recipient vector dephosphorylation, ligation, and transformation, and colony PCR procedures were the same as done for CFP, but colony PCR primers were as follows: forward 5′ CCTGTGTCAGTGCAAGGTGT 3′, reverse 5′ TTCCCCAAAGCAGA-AGAAGA 3′. Products were also visualized on a 1% agarose gel. A positive colony was grown up and plasmid DNA isolated the same way as for CFP. Confirmation of plasmid sequence was done at GENEWIZ (South Plainfield, NJ, USA) with the forward and reverse primers.

### Generation of Luciferase Reporter Constructs

We obtained another plasmid with the non-risk IRF5 promoter (NM_002200) from Genecopoeia (HPRM19470-PG02). To obtain the promoter with the risk allele, a PCR was performed on donor genomic DNA with restriction sites for EcoRI and HindIII in the product. The primers used were as follows: forward 5′ TCTTGGAATTCCCCTCCTGTTTTCCTTCCCTGCTAT 3′, reverse 5′ GCCA-ACCTGCCGGGCACT 3′. The reaction used 20 ng DNA and the same reagents and concentrations as the PCR described in Section “Genotyping,” but final concentrations of DMSO, MgCl_2_, and primers were 3.5%, 1.5 mM, and 0.5 µM, respectively. The cycling conditions were 95°C for 5 min, 38 cycles of 95°C for 20 s, 63°C for 30 s, 72°C for 1 min, and 1 cycle of 72°C for 5 min. Both the donor plasmid and PCR product were restriction digested with EcoRI and HindIII; the donor plasmid was then dephosphorylated, ligated with the product, transformed into *E. coli*, grown and isolated the same way as described in “Generation of IRF5 and CPF-overexpressing constructs.” At this point we had two plasmids; one with the non-risk allele and one with the risk allele. We also obtained pGL4.10 promoterless *Firefly* and pRL thymidine kinase (TK) promoter-driven *Renilla* luciferase vectors (Promega). We digested the promoterless firefly vector, the non-risk allele plasmid, and the risk allele plasmid with BglII and HindIII (New England Biolabs), dephosphorylated the donor *Firefly* vector, and ligated it with either the purified non-risk or risk allele insert. Ligation products were transformed into *E. coli* and selected based on carbenicillin resistance. Colonies were picked and screened *via* colony PCR using the same primers as in Section “Genotyping.” The colony PCR was done with the same reagents and concentrations as in Section “Genotyping,” but cycling conditions were as follows: 95°C for 10 min, 40 cycles of 95°C for 20 s, 56°C for 30 s, 72°C for 1 min, and 1 cycle of 72°C for 5 min. Products were visualized on a 1% agarose gel. A successful colony was transformed into *E. coli*, grown and isolated the same way as described in “Generation of IRF5 and CPF-overexpressing constructs.” The resulting non-risk allele *Firefly* vector and risk allele *Firefly* vector were confirmed by sequencing at GENEWIZ with the forward primer used in Section “Genotyping.” Mutation of the risk allele in the *Firefly* vector to a non-SP1 binding site was done with the QuikChange II Site-Directed Mutagensis Kit according to manufacturer’s instructions. The mutagenic primers used were as follows: forward 5′ GGGCGGGGCGGTTCCGGGCAC-TGCCC 3′, reverse 5′ GGGCAGTGCCCGGAACCGCCCCGCCC 3′.

### Transient Transfection and IRF5 Overexpression

Raji and Daudi cells were transfected with 4 µg plasmid DNA using the Amaxa Nucleofector Kit V and 3 µg DNA using the Amaxa Nucleofector™ Kit L (Lonza), respectively, according to the manufacturer’s instructions. Half the amount of DNA in each cotransfection reaction consisted of the CFP reporter vector, and the resultant amount consisted of either IRF5 overexpression vector or empty vector. To control for plasmid size, IRF5 overexpression vector or empty vector were transfected in equimolar amounts. Transfected cells were incubated in culture medium for 24 h at 37°C in a humidified 5% CO_2_ incubator. For experiments involving stimulation of transfected cells with IRF5 activating agents, cells were placed into culture medium with 2.5 µM CpG or 5 µg/ml R848 immediately after transfection.

### Luciferase Reporter Gene Assay

The following table illustrates transfection conditions for the given cell lines:

**Table:** 

Cell line	μg DNA	Nucleofector™ Cell Line Kit and program	Ratio firefly:renilla	Incubation time (h)
Ramos	4	V, O-06	10:1	16
Raji	4	V, M-13	10:1	16
Daudi	3	L, A-20	10:1	16
Jurkat	2	V, X-01	10:1	16
THP-1	2	V, V-01	2:1	13

Transfected cells were incubated in culture medium for 24 h at 37°C in a humidified 5% CO_2_ incubator. After incubation, firefly and renilla activity was measured using the Dual-Glo^®^ Luciferase Assay System (Promega) according to the manufacturer’s instructions. Four independent experiments were done with all five cell lines comparing the risk and non-risk promoters; three independent experiments were done with THP-1 cells comparing the risk, non-risk, and mutated promoters.

### Electro-Mobility Shift and Supershift Assays

Ramos, Raji, Daudi, and THP-1 cells were subcultured at 4 × 10^5^/ml and harvested when a total of 35 × 10^6^ cells was present (~48 h later). Nuclear extract was prepared using the NE-PER^®^ Nuclear and Cytoplasmic Extraction Kit (ThermoFisher Scientific) according to the manufacturer’s instructions. Infrared (700 nm) labeled oligonucleotides were obtained from Integrated DNA Technologies (Coralville, IA, USA). Probe sequences used were as follows: non-risk 5′ AGTGGATTCGCGGGG-CGGGGCGGGGCACTGC 3′, risk 5′ AGTGGATTCGCGGGGCGGGGCGGGGCGGGGCACT-GC 3′. The Odyssey Infrared electro-mobility shift assay (EMSA) Kit (LI-COR) was used according to the manufacturer’s instructions. For supershift reactions, 1 µg rabbit anti-SP1 or isotype control (Cell Signaling Technology) was used. Gels were imaged on a LI-COR Infrared Odyssey machine.

### Statistical Analysis

Statistical analyses were performed with GraphPad Prism 6 software. Statistical significance was determined with the following tests where indicated: Friedman test with Dunn’s multiple comparison test, Wilcoxon matched-pairs signed rank test, Mann–Whitney test, and the Kruskal–Wallis test. *p* Values < 0.05 were considered significant.

## Results

### Common IRF5 Risk and Non-Risk Haplotypes Are Present Across Various Populations

**Figure 1:**
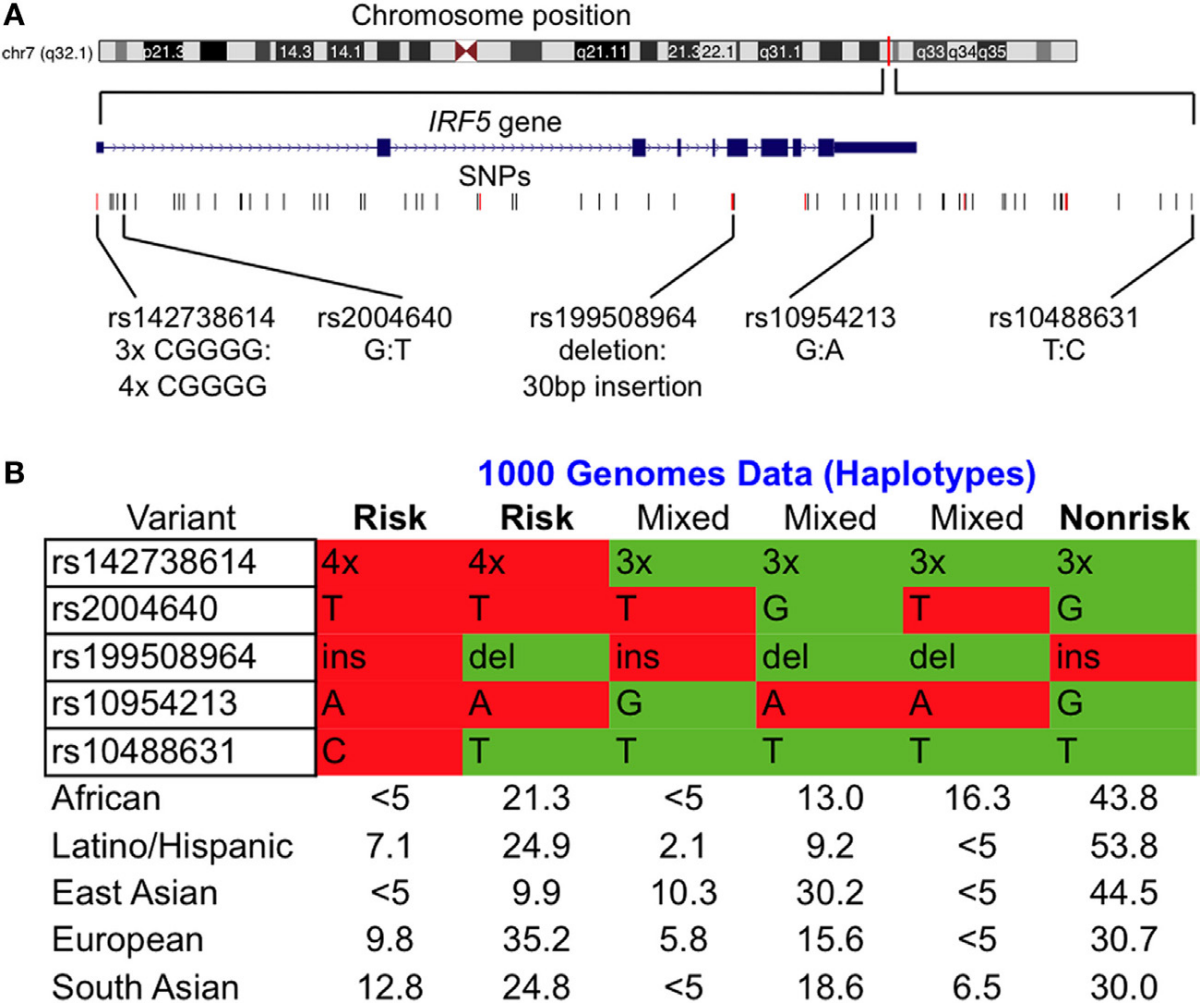
Common interferon regulatory factor 5 (IRF5) risk and non-risk haplotypes are present across various populations. **(A)** Chromosomal location of the IRF5 locus of interest with SNPs considered in our study. Alleles are indicated as non-risk:risk. **(B)** Condensed haplotypes obtained from 1000 Genomes present in >5% of the indicated ethnic populations. Those labeled risk and non-risk were included in our study. Abbreviations: ins, insertion; del, deletion.

Using the 1000 Genomes Project data (36), we determined haplotype frequencies for the IRF5 locus (Figure 1A) for AFRs, Latino/Hispanics, EASs, EURs, and SASs Most of the IRF5 variants associated with SLE are in a high degree of LD (Figure 1B). In all populations, the risk variants of rs142738614, rs2004640, rs10954213, and rs10488631 are inherited together, with the exception of the risk allele of rs10488631 which is present at a frequency <5% in AFRs and EASs. Our data show that (1) rs142738614, rs2004640, and rs10954213 are three suitable markers for determining the presence of the common risk and non-risk haplotype across populations and (2) four copies of the CGGGG sequence of the rs142738614 promoter indel is a suitable proxy for the risk haplotype.

### IRF5 Expression Is Higher in Monocytes and IgD^−^ Memory B Cell Subsets Independent of Haplotype

**Figure 2:**
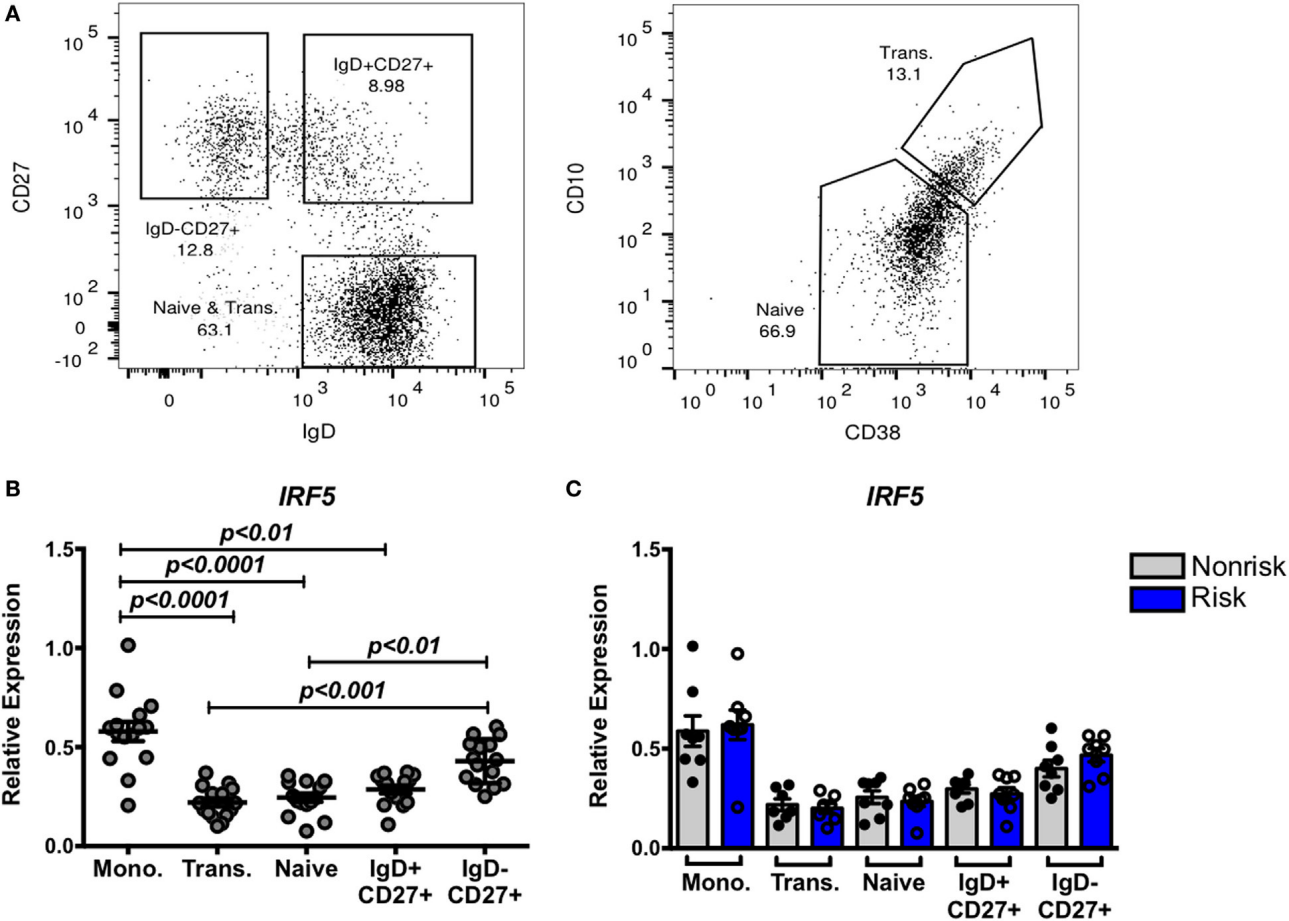
Interferon regulatory factor 5 (IRF5) expression is higher in monocytes and IgD^−^ memory B cell subsets independent of haplotype. Monocytes and four subsets of B cells were sorted after gating on CD14^+^ and CD19^+^ cells, respectively. **(A)** Gating strategy for IgD^+^CD27^+^, IgD^−^CD27^+^, CD27^−^CD10^lo^CD38^l0^ (naive), and CD27^−^CD10^hi^CD38^hi^ (transitional) B cells. Sorted cells were subjected to qPCR for IRF5 **(B,C)**, and results are shown combined **(B)** and separated by haplotype **(C)**. *p* < 0.01 by Friedman test with Dunn’s multiple comparisons test **(B)**, *p* = ns by Mann–Whitney test for each B cell subset **(C)**.

In SLE, B cell tolerance checkpoints are compromised (37), and it has been shown that SLE risk alleles can affect those tolerance checkpoints. To address whether the IRF5 risk haplotype might affect B cell tolerance, we first asked whether IRF5 expression differs across B cell subsets and whether the risk and non-risk haplotypes are differentially regulated as expression quantitative trait loci (eQTL). We compared expression of IRF5 in B cells alongside expression of IRF5 in monocytes. We sorted monocytes based on expression of CD14 and gated B cells based on CD19 expression. CD19^+^ B cells were sorted into four populations: naïve (IgD^+^CD27^−^CD10^lo^CD38^lo^), transitional (IgD^+^CD27^−^CD10^hi^CD38^hi^), and CD27^+^ memory B cells, both IgD^+^CD27^+^ and IgD^−^CD27^+^ (Figure 2A). IRF5 expression was significantly higher in monocytes than in B cells, and highest in CD27^+^IgD^−^ B cells out of all the sorted B cell subsets (Figure 2B). Whether or not B cells or monocytes were from subjects carrying the risk or non-risk haplotype did not affect gene expression (Figure 2C). Thus, although IRF5 may play an important role in class switched, antigen-experienced B cells, the presence of the IRF5 risk haplotype does not alter gene expression in unstimulated, circulating B cells or unstimulated monocytes.

### IRF5 Risk Alleles Do Not Differentially Affect IRF5 Expression by *In Vitro* Stimulated B Cells

**Figure 3:**
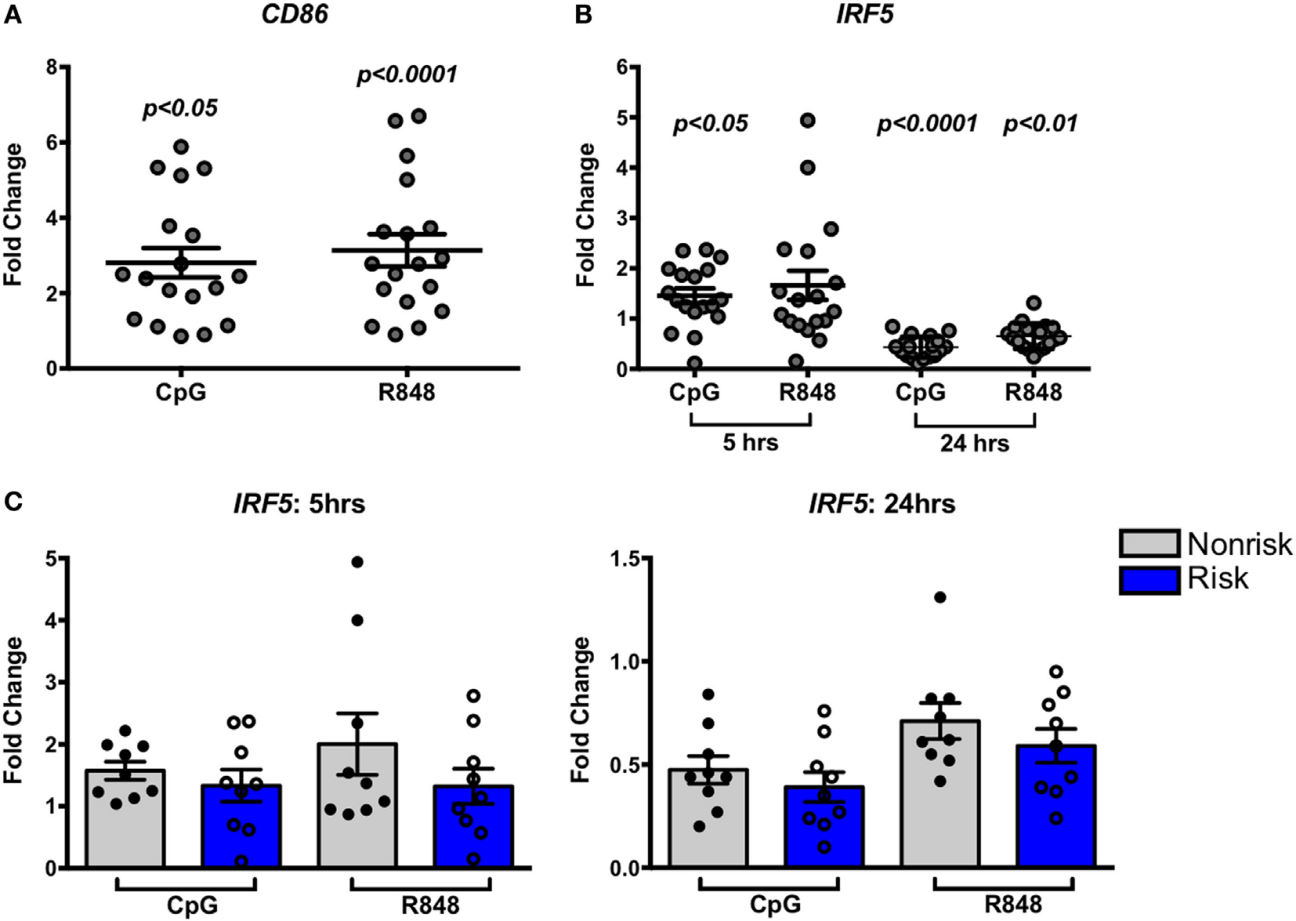
Interferon regulatory factor 5 (IRF5) risk alleles do not affect induced IRF5 expression in B cells. Purified B cells were stimulated with toll-like receptor agonists CpG or R848 for the indicated times and subjected to qPCR for CD86 **(A)** or IRF5 **(B,C)** IRF5 qPCR. Results are shown combined **(B)** and separated by haplotype **(C)**. Unstimulated cell expression values are normalized to 1.0. *p* < 0.05, *p* < 0.01, and *p* < 0.0001 by Friedman test with Dunn’s multiple comparisons test **(A,B)**, *p* = ns by Wilcoxon matched-pairs signed rank test for each treatment **(C)**.

The impact of some disease-associated risk haplotypes is seen only in activated cells as “response eQTLs.” We therefore asked whether stimulation of B cells through an IRF-dependent pathway would trigger haplotype-specific expression differences. B cells were stimulated with the TLR9 and TLR7 agonists, CpG or R848, respectively, for 5 or 24 h and IRF5 gene expression was measured. Successful B cell stimulation was confirmed by increased CD86 expression after 5 h (Figure 3A). We observed a slight but significant increase in IRF5 expression 5 h after CpG stimulation as well as a decrease in expression 24 h after CpG or R848 stimulation (Figure 3B) confirming that IRF5 is within the TLR7 and 9 pathways. The presence of the IRF5 risk or non-risk haplotype did not differentially affect the response to TLR agonists (Figure 3C).

### Susceptibility to Apoptosis in B Cells Does Not Differ According to IRF5 Haplotype

**Figure 4:**
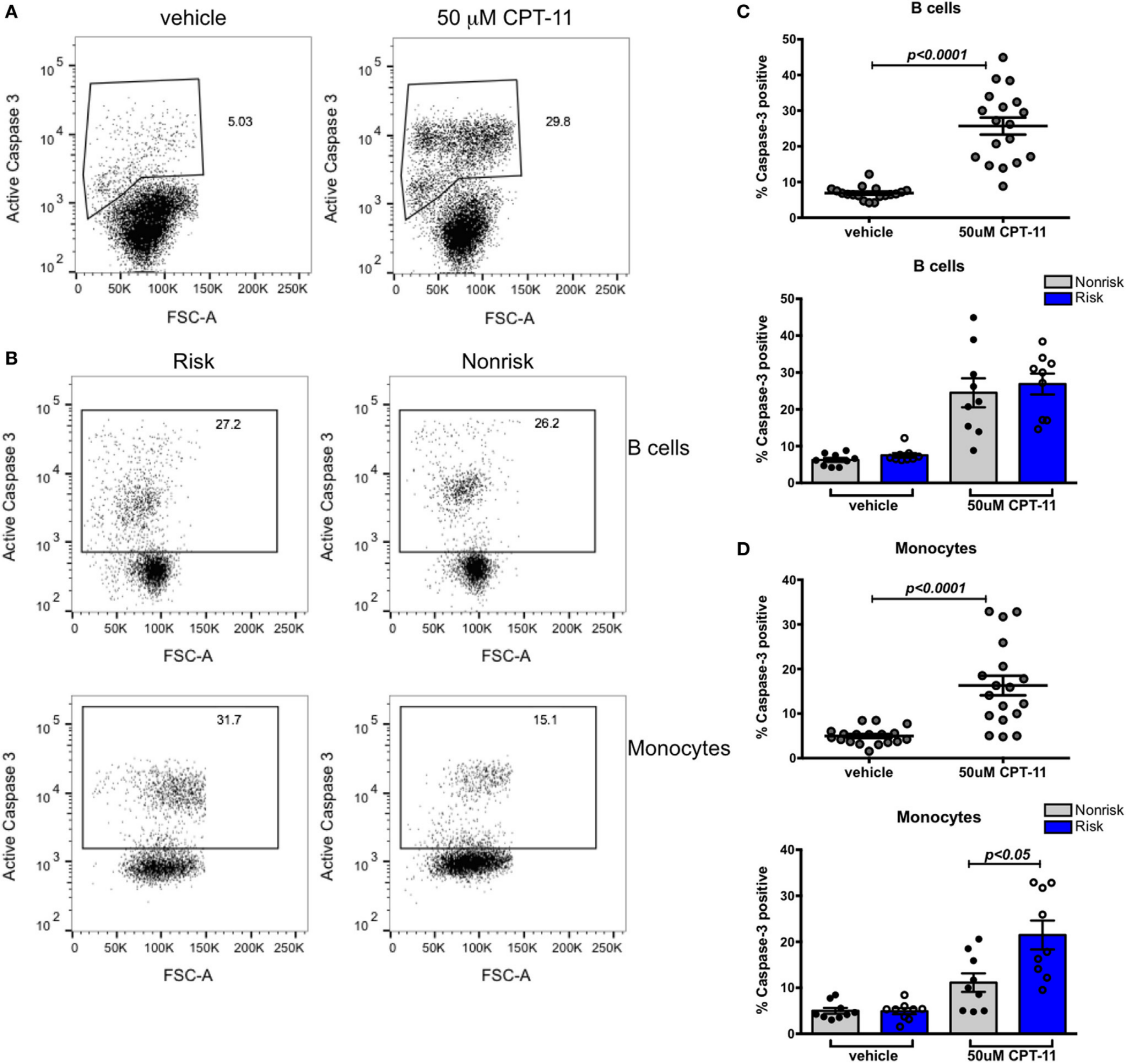
Susceptibility to apoptosis in B cells does not differ according to interferon regulatory factor 5 haplotype. Fresh peripheral blood mononuclear cells (PBMCs) were treated with 50 mM CPT-11 for 8 h, stained for CD19, CD14, viability dye, active caspase 3, and analyzed by flow cytometry. **(A)** Representative dot plots of vehicle and CPT-11-treated PBMCs. **(B)** Representative dot plots showing proportions of caspase 3^+^ B cells (top row) and monocytes (bottom row) in risk (left column) and non-risk (right column) haplotype subjects. **(C)** Proportion of apoptotic CD19^+^ cells shown combined (top) and separated by haplotype (bottom). **(D)** Apoptotic CD14^+^ monocytes shown combined (top) and separated by haplotype (bottom). *p* < 0.0001 by Wilcoxon matched-pairs signed rank test [**(C,D)**, top], *p* = ns by Mann–Whitney test for each treatment [**(C)**, bottom], *p* < 0.05 by Mann–Whitney test for each treatment [**(D)**, bottom].

While we observed no significant difference in basal or stimulated levels of expression of IRF5, we thought it important to ask whether an effect of the IRF5 risk haplotype might be revealed in a more sensitive bioassay. IRF5 is known to play a critical role in cell cycle arrest and cell death (7). Irinotecan (CPT-11), a potent topoisomerase inhibitor and DNA damaging agent, activates an IRF5-dependent apoptotic program (38). We asked whether IRF5 haplotypes differentially affect apoptosis induced by CPT-11. PBMCs from healthy genotyped donors were treated with 50 µM CPT-11 or vehicle for 8 h and analyzed for the expression of active caspase 3 by flow cytometry. CPT-11 induced a significant increase in caspase 3 positive PBMCs (Figure 4A). The effect of CPT-11 on inducing active caspase 3 was evident in both B cells (CD19^+^) and monocytes (CD14^+^) (Figures 4C,D, top). Interestingly, there was a haplotype dependent induction of apoptosis in monocytes; risk haplotype carriers had a higher proportion of cells undergoing apoptosis than non-risk haplotype carriers (Figures 4B,D, bottom). This haplotype dependent induction of apoptosis was not evident in B cells (Figures 4B,C, bottom). These findings suggest that although IRF5 plays a role in apoptosis in multiple cell types, the SLE risk haplotype enhances IRF5-mediated apoptosis in monocytes and not in B cells.

### IRF-Specific B Cell Stimulation Results in Upregulated CD86 Expression and ASC Differentiation

**Figure 5:**
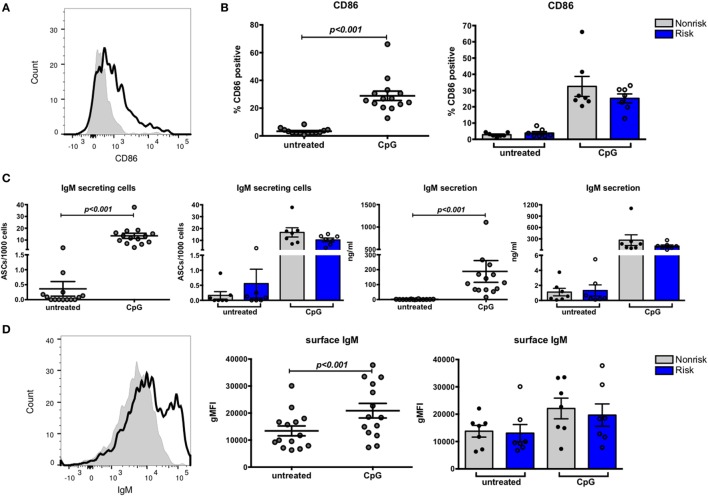
B cell stimulation results in upregulated CD86 expression and antibody-secreting cell (ASC) differentiation. Naive B cells were cultured for 4 days with or without CpG and analyzed by flow cytometry. **(A)** Representative histogram of CD86 expression on untreated (shaded peak) and CpG-treated (black outlined peak) B cells. **(B)** CD86 expression on B cells combined (left) and separated by haplotype (right). **(C)** ELISpot data showing proportion of ASCs combined (far left) and separated by haplotype (center left) and ELISA data showing IgM secretion by B cells combined (center right) and separated by haplotype (far right). **(D)** Representative histogram of surface IgM expressed on untreated (shaded peak) and CpG-treated (black outlined peak) B cells. Flow cytometric results of IgM expression on B cells combined (center) and separated by haplotype (right). *p* < 0.001 by Wilcoxon matched-pairs signed rank test [**(B)**, left; **(C)**, far left and center right; **(D)**, center], *p* = ns by Mann–Whitney test for each treatment [**(B)**, right; **(C)**, center left and far right; **(D)**, right].

Based on murine studies, IRF5 has been shown to play a role in heavy chain class switch recombination to IgG (17, 18) and in plasma cell differentiation which requires upregulation of the transcription factor Blimp1 (15); IRF5 controls Blimp1 expression. We were interested in determining whether IRF5 haplotypes differentially promote class switching and plasma cell differentiation in human B cells. Naïve B cells were cultured with or without CpG for 4 days and subjected to multiple functional assays including ELISpot, ELISA, and flow cytometry. B cell activation was demonstrated by upregulation of CD86 (Figures 5A,B, left), but the degree of upregulation did not differ according to haplotype (Figure 5B, right). ELISpot analysis indicated that CpG induced B cells to differentiate into IgM ASCs (Figure 5C, far left). We confirmed IgM secretion by ELISA (Figure 5C, center right). CpG treated cells also exhibited increased surface IgM (Figure 5D, left and center). In all these analyses, results did not differ according to IRF5 haplotype (Figure 5C, center left and far right; Figure 5D, right).

**Figure 6:**
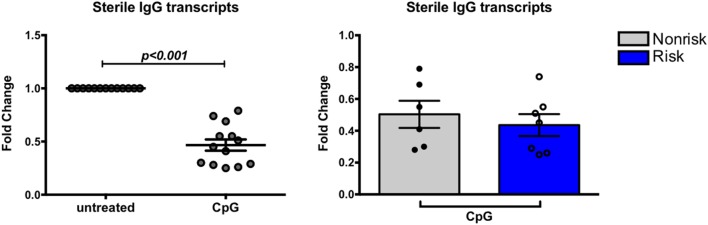
Expression of sterile transcripts in B cells. B cells were cultured for 24 h with or without CpG and subject to qPCR for germline IgG. Results are shown combined (left) and separated by haplotype (right). *p* < 0.001 (left) by Wilcoxon matched-pairs signed rank test, *p* = ns by Mann–Whitney test (right).

IgG secreting cells or surface IgG^+^ cells were not detected after the 4-day culture (data not shown), suggesting CpG alone may not be sufficient to induce class switching to IgG or that 4-day cultures are too short term for production of IgG to be evident. We therefore decided to perform quantitative PCR to measure the relative abundance of IgG germline mRNA in naïve B cells cultured with or without CpG for 24 h. Transcription of germline constant region mRNA is reported to precede isotype switching (39); the resulting transcripts are not translated into protein and are therefore termed “sterile transcripts.” We considered that we might detect these sterile transcripts before we could detect IgG proteins. Surprisingly, we observed a decrease in abundance of sterile transcripts (Figure 6) and this decrease was observed equally in risk and non-risk haplotype B cells, suggesting that TLR7 and 9 signaling may not be sufficient for class switch recombination. Overall, we found that CpG stimulation upregulates CD86 and surface IgM on B cells and transforms naïve B cells into IgM secreting cells, and these effects occur independent of IRF5 haplotype.

### IRF5 Does Not Affect FcγRIIb Expression in B Cells

**Figure 7:**
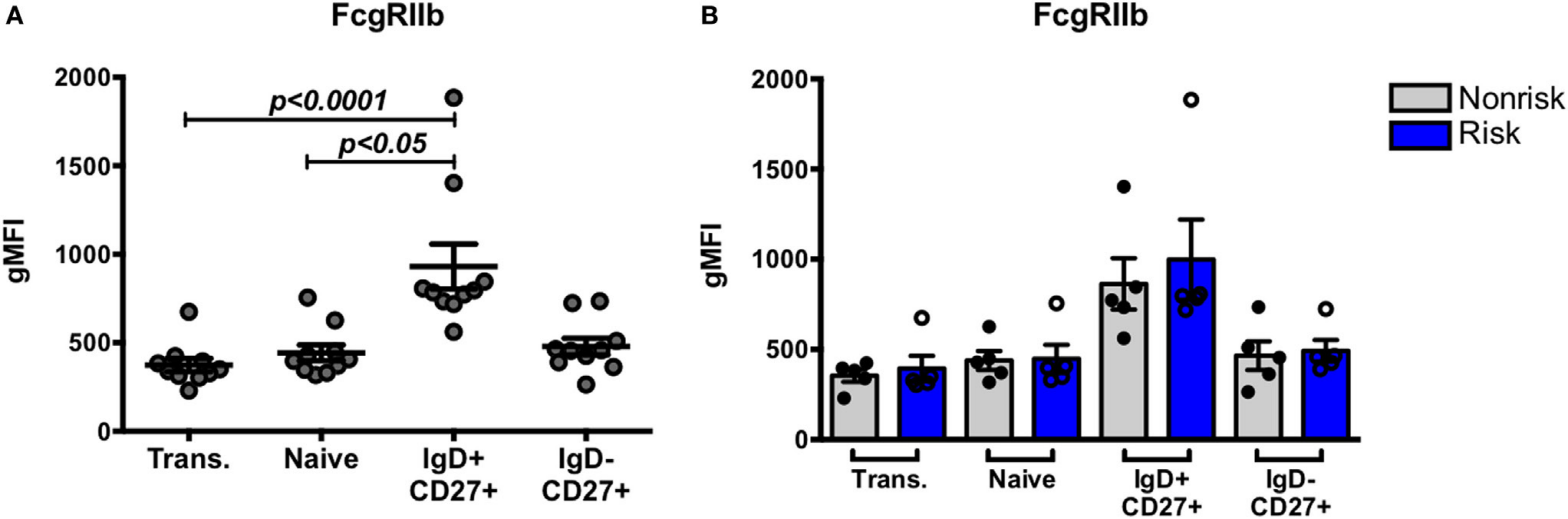
Interferon regulatory factor 5 risk alleles do no affect baseline FcγRIIb expression in B cell subsets. B cell subsets were analyzed similarly as in Figure 1. **(A)** Flow cytometric results of FcγRIIb protein expression in combined B cell subsets and **(B)** separated by haplotype. *p* < 0.05 and *p* < 0.0001 by Friedman test with Dunn’s multiple comparisons test **(A)**, *p* = ns by Mann–Whitney test for each B cell subset **(B)**.

The expression of FcγRIIb on B cells represents an important B cell tolerance mechanism. Triggering FcγRIIb serves as one mechanism to limit B cell activation through delivery of inhibitory signals that contribute to regulation of the immune response (22). SLE patients fail to upregulate FcγRIIb on CD27^+^ memory B cells (40). Studies with mouse models suggest the existence of crosstalk between FcγRIIb and IRF5 with negative regulation of FcγRllb by IRF5 (20). We asked whether a relationship between IRF5 and FcγRIIb exists in human B cells, and if the IRF5 risk haplotype might be associated with a decrease in FcγRllb expression. Expression of FcγRIIb was measured on B cell subsets from genotyped subjects using flow cytometry. Overall, FcγRIIb expression was highest on IgD^+^CD27^+^ B cells (Figure 7A). IRF5 haplotype did not differentially affect FcγRIIb expression in any B cell subset (Figure 7B). These results show that the IRF5 risk haplotype does not affect expression of FcγRIIb on B cells.

**Figure 8:**
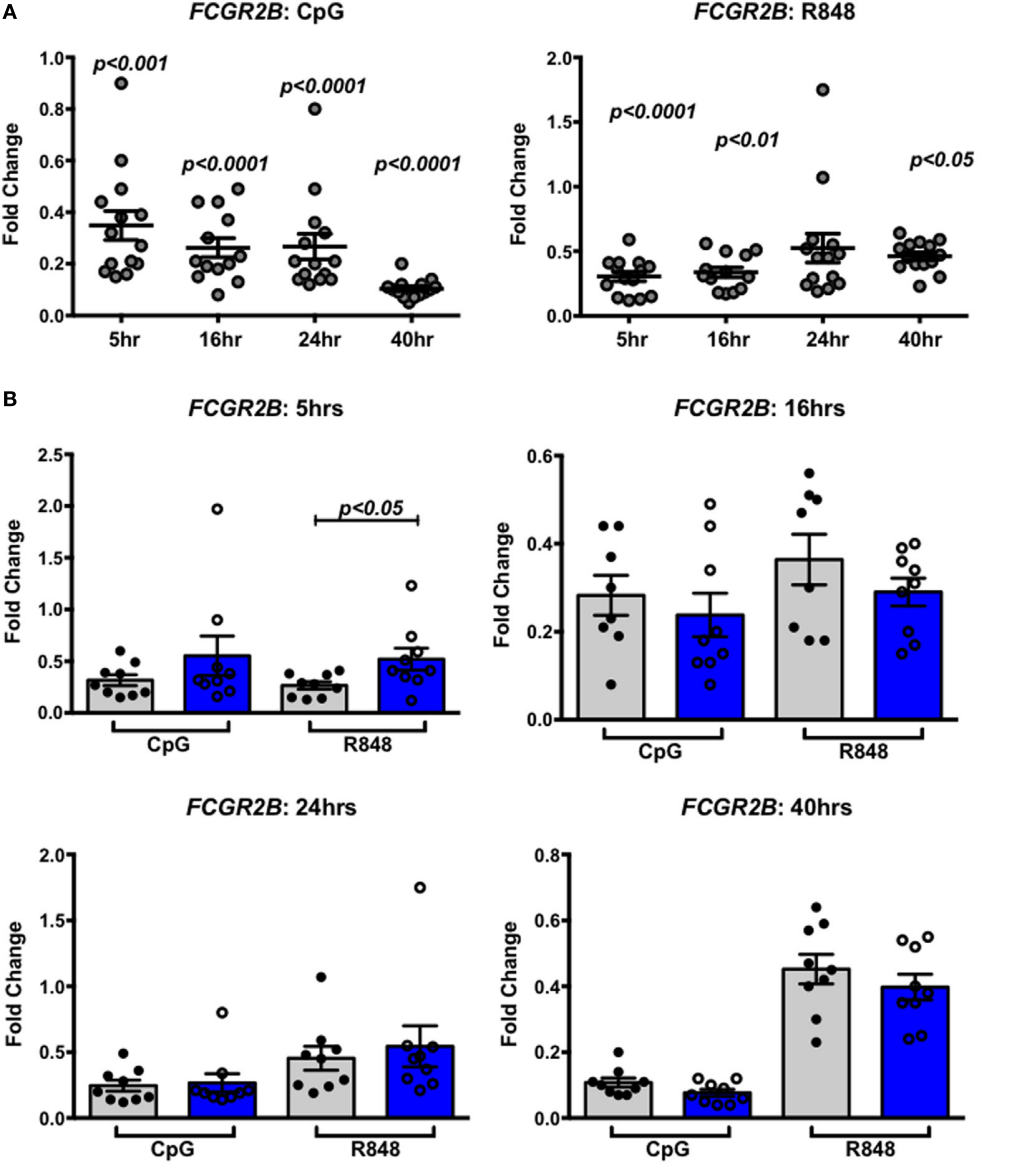

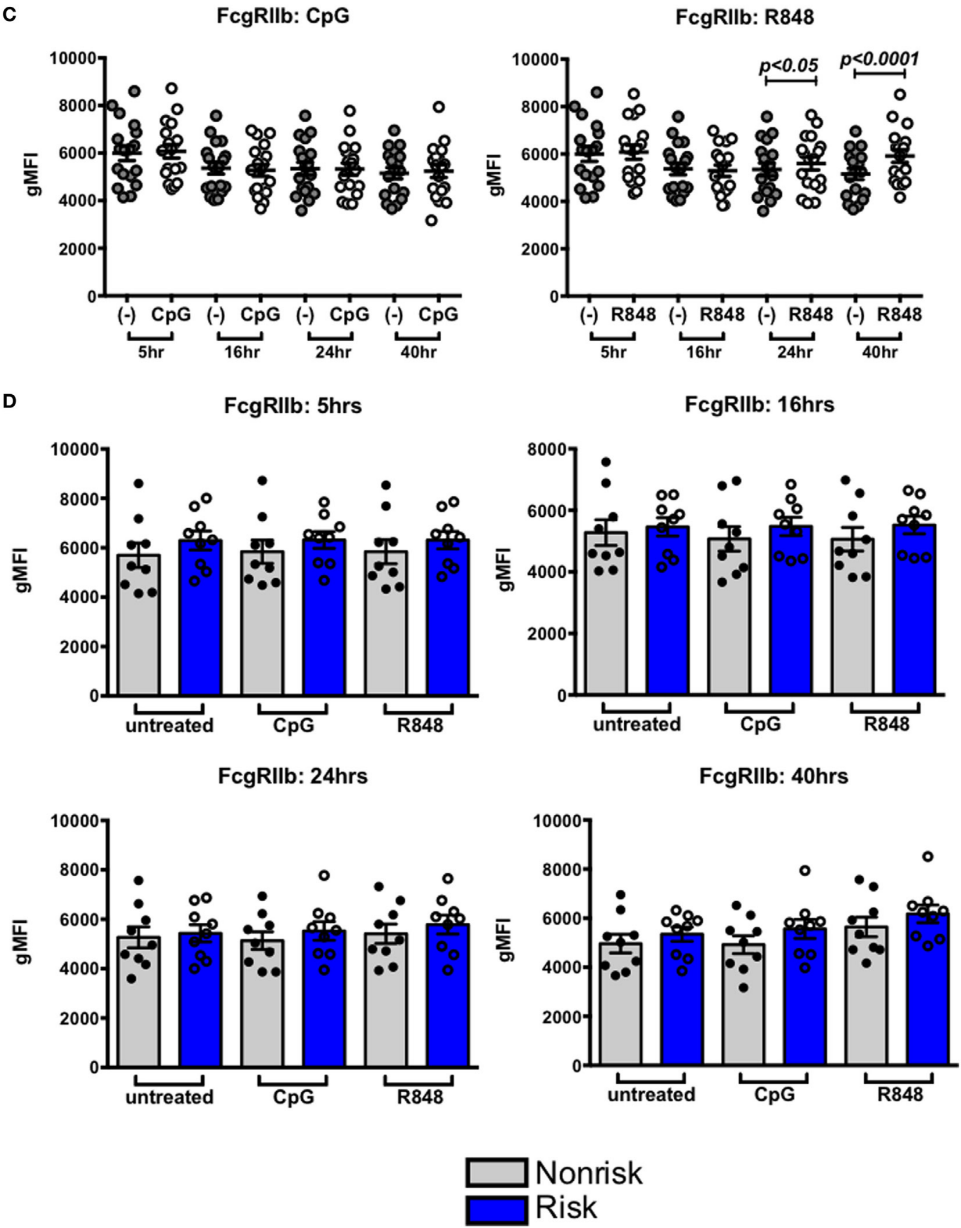
Interferon regulatory factor 5 activation does not affect FcγRIIb expression in B cells. **(A,B)** FcγRIIb mRNA expression in B cells treated with CpG (right) or R848 (left) for the indicated times. Results are combined **(A)** and separated by haplotype **(B)**. **(C,D)** FcγRIIb protein expression in B cells treated with CpG (right) or R848 (left) for the indicated time. Results are combined **(C)** and separated by haplotype **(D)**. All unstimulated cell expression values are normalized to 1.0. *p* < 0.05, *p* < 0.01, *p* < 0.001, and *p* < 0.0001 by Friedman test with Dunn’s multiple comparisons test **(A,C)**, *p* = ns by Mann–Whitney test for each treatment **(B,D)**.

**Figure 9:**
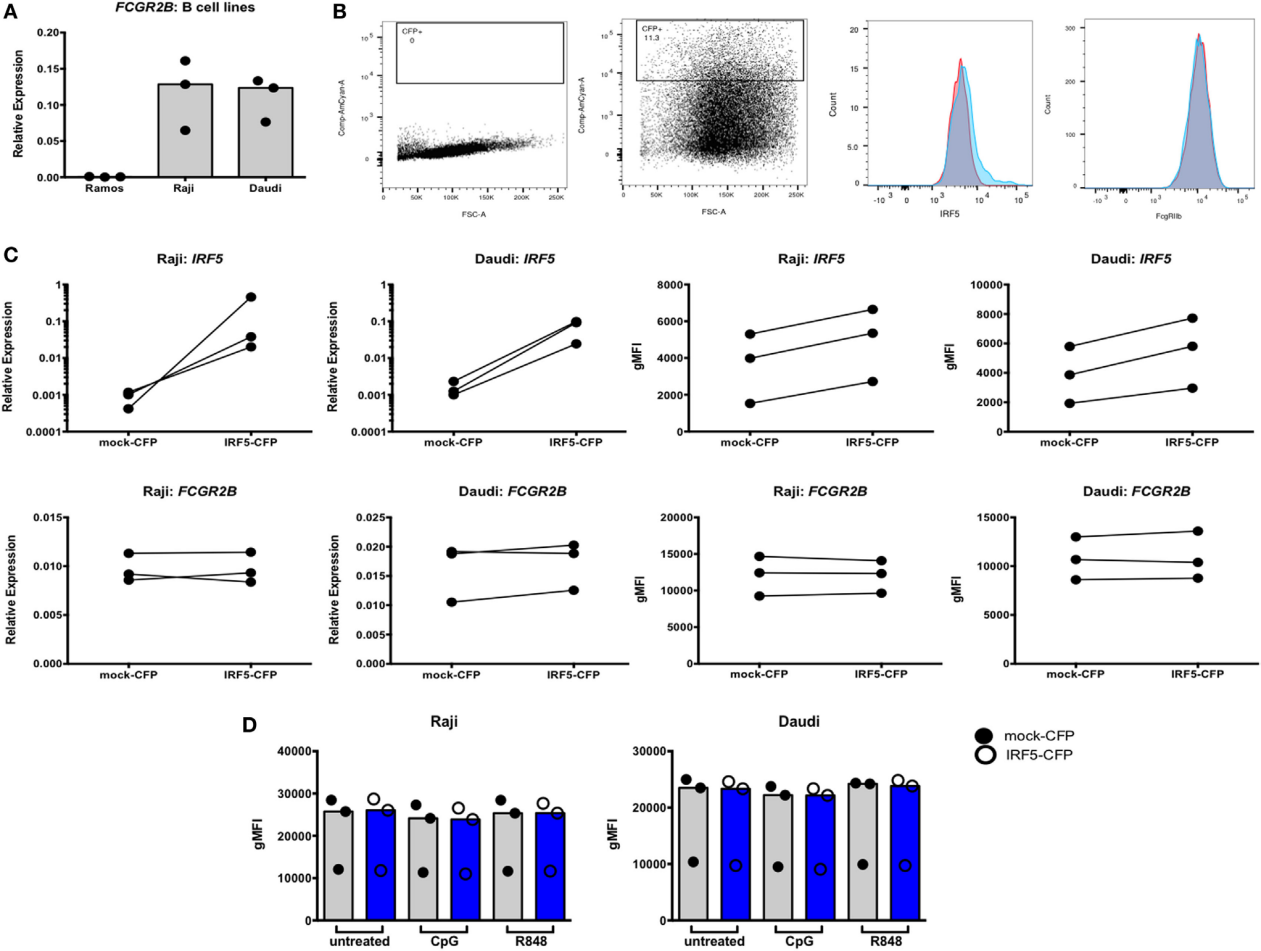
Interferon regulatory factor 5 (IRF5) overexpression does not affect FcγRIIb expression in Raji and Daudi cells. **(A)** FcγRIIb expression in Ramos, Raji, and Daudi cells. **(B)** Representative flow cytometric data showing untransfected (far left) and transfected (center left) cells. Gated cells were used for sorting and analyses. Representative histogram of IRF5 protein expression on mock (red) and IRF5 (blue) transfected cells (center right), representative histogram of FcγRIIb protein expression on mock (red) and IRF5 (blue) transfected cells (far right). **(C)** IRF5 mRNA (top row, far left and left) and protein (top row, far right and right) levels in CFP^+^ cells. FcγRIIb mRNA (bottom row, far left and left) and protein (bottom row, far right and right) levels in CFP^+^ cells. **(D)** FcγRIIb protein levels in CFP^+^ cells stimulated with CpG or R848 for 24 h. All data shown are representative of three independent experiments.

We next asked whether IRF-dependent stimulation of B cells would result in haplotype-specific differences in FcγRIIb expression. B cells were stimulated with CpG or R848 for up to 40 h, and FcγRIIb transcript and protein expression were measured by qPCR and flow cytometry, respectively. CpG and R848 stimulation both resulted in significant decreases in FcγRIIb mRNA expression as early as 5 h after stimulation (Figure 8A). Surprisingly, protein expression remained unaffected and increased slightly at 24 and 40 h after R848 stimulation (Figure 8C). Although statistically significant, the changes in protein we observed in R848 stimulated B cells were very small and it is uncertain whether changes of this magnitude are enough to affect the biology of B cells. There were no haplotype-specific effects on mRNA or protein levels (Figures 8B,D). Considering IRF5 expression is low in B cells, we next sought to determine whether overexpressing IRF5 in B cells would have a measureable effect on expression of FcγRIIb. We hypothesized that overexpressing IRF5 would result in a decrease in FcγRIIb, similar to the negative regulation seen in mice (20). First we assessed expression of FcγRIIb in the Ramos, Raji, and Daudi human B cell lines (Figure 9A). We chose to utilize Raji and Daudi cells, as both expressed significant levels of FcγRllb. We cotransfected Raji and Daudi cells with a CFP reporter plasmid and either an IRF5 or empty (mock) plasmid. We isolated CFP^+^ cells (Figure 9B, center left) and analyzed IRF5 transcript (Figure 9C, top center left and far left) and protein (Figure 9B, center right and Figure 9C, top center right and far right) and FcγRIIb transcript (Figure 9C, bottom center left and far left) and protein (Figure 9B, far right and Figure 9C, bottom center right and far right) levels. We confirmed an increase in IRF5 mRNA and protein, but expression of FcγRIIb RNA and protein was unchanged.

To determine whether stimulation of the transfected cells was necessary to activate the overexpressed IRF5, transfected cells were stimulated with CpG or R848 for 24 h and analyzed by flow cytometry. Activation of overexpressed IRF5 did not affect FcγRIIb expression (Figure 9D). These findings show that overexpressing or activating IRF5 does not affect FcγRIIb expression in human B cells.

### The IRF5 Promoter Risk indel Results in Increased SP1 Binding and IRF5 Transcription in Myeloid Cells

**Figure 10:**
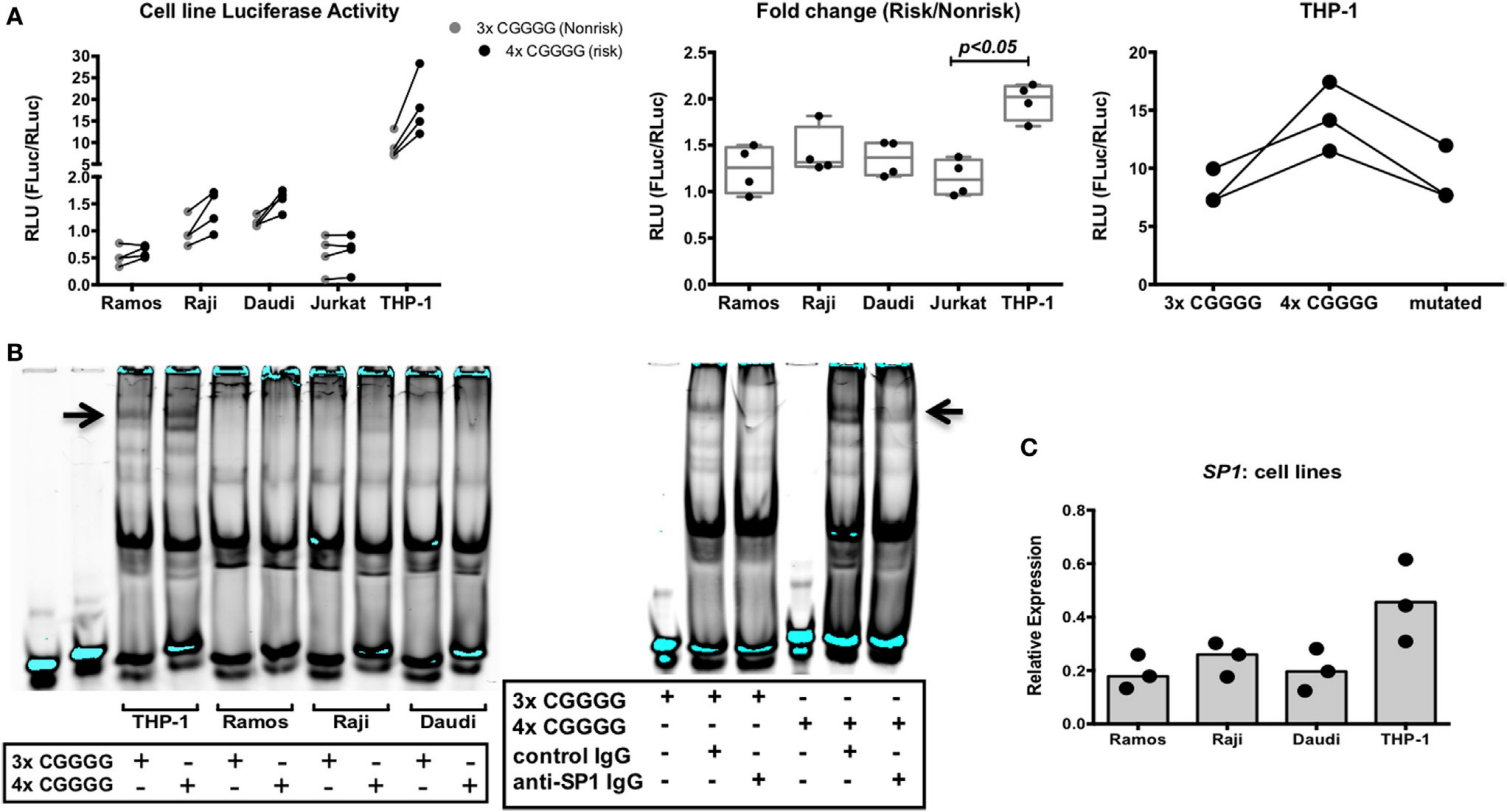
The interferon regulatory factor 5 (IRF5) promoter risk insertion/deletion (indel) results in increased IRF5 transcription and specificity protein 1 (SP1) binding in myeloid cells. **(A)** Normalized activities of non-risk and risk indel promoter-driven luciferase transcription in various cell lines (left), fold change in luciferase activity due to the risk indel in various cell lines (center), and risk, non-risk, and mutated promoter-driven luciferase transcription in THP-1 cells (right). Results of three or four independent experiments are shown. **(B)** Electrophoretic mobility shift assay showing binding to the 3× and 4× CGGGG indel by various nuclear extracts (left), supershift assay showing SP1 binding to the 3× and 4× CGGGG indel in nuclear extracts of THP-1 cells (right). Results of three independent experiments are shown. **(C)** SP1 mRNA levels in various cell lines. Results of three independent experiments are shown. *p* < 0.05 by Kruskal–Wallis test [**(A)**, center].

We decided to assess the strength of the IRF5 promoter containing either three or four repeats of the 5 bp CGGGG sequence in various cell lines: myeloid, T cell, and B cell lines. Previous work has shown increased IRF5 expression with the risk indel in *ex vivo* PBMCs and in HEK293 cells using a minigene assay (32). We created two *Firefly* luciferase reporter plasmids; one with the three repeats of CGGGG and one with four repeats, and used a normalizing *Renilla* luciferase plasmid under control of the constitutive HSV-TK promoter. Ramos, Raji, Daudi, Jurkat, and THP-1 cells were transfected and analyzed for relative luciferase light units (Figure 10A, left). The basal level of IRF5 transcription is highest in THP-1 cells. The fold change in IRF5 transcription was also highest in THP-1 cells, as was induction of IRF5 transcription (Figure 10A, center).

Four repeats of the 5 bp CGGGG sequence in the risk haplotype introduce another binding site for the transcription factor SP1 (32). We asked whether SP1 binding to this additional site has a role in increased transcription of IRF5. To this end, we created an additional 4× indel IRF5 promoter-*Firefly* luciferase plasmid, with one SP1 binding site mutated to a non-SP1 binding site. We transfected THP-1 cells with the mutated plasmid and compared the results to the 3× and 4× indel promoter reporter plasmids. We hypothesized that if SP1 were important for the observed increased IRF5 transcription seen with the 4× indel, abolishing the additional binding site would result in luciferase readings similar to those obtained with the 3× promoter reporter plasmid. Interestingly, this result was obtained in THP-1 cells (Figure 10A, right). Thus, the SP1 transcription factor has a role in the increased transcription of the IRF5 risk haplotype observed in myeloid cells.

We next decided to assess binding of nuclear transcription factors from B cell lines and THP-1 cells to the 3× and 4× indel. Cell lysates were incubated with infrared-labeled oligonucleotides containing three or four repeats of CGGGG and analyzed by EMSA. A specific band was observed when the 4× indel was incubated with THP-1 nuclear extracts compared with extracts of the B cell lines (Figure 10B, left). The banding pattern was also present in Raji extract with the 4× indel, albeit very faintly. To ask whether the binding pattern observed in THP-1 cells was due to SP1, we performed a supershift EMSA with anti-SP1 antibody. Interestingly, we observed a change in the pattern when cell lysates were incubated with anti-SP1 (Figure 10B, right). We then asked if this is due to higher IRF5 and SP1 expression in THP-1 cells compared with B cell lines. We observed that both IRF5 and SP1 (Figure 10C) mRNA levels were higher in THP-1 cells than in B cell lines. The data suggest that the differential expression of SP1 in monocytes and B cells is responsible for the haplotype-specific increased expression of IRF5 observed in myeloid cells and not in B cells.

## Discussion

Approximately 100 genes have been identified in GWAS of SLE (41); however, the functionality of the majority of the risk alleles has not been elucidated. The goal of this study was to define an SLE-associated IRF5 risk haplotype and determine the effects of the risk haplotype on the biology of B cells. We first defined a risk haplotype common to various populations that included the 4× CGGGG indel in the promoter region and two risk SNPs, rs10954213 and rs2004640. Our major findings were (1) the IRF5 risk haplotype does not affect quantitative IRF5 expression in resting or activated B cells, (2) IRF5 activation triggers CD86 upregulation and differentiates B cells into IgM ASCs, but this is not influenced by IRF5 haplotype, (3) IRF5 does not regulate FcγRIIb in human B cells, (4) the IRF5 risk haplotype differentially affects IRF5-mediated apoptosis in monocytes and not in B cells, and (5) the transcription factor SP1 binds more strongly to the 4× CGGGG indel in myeloid cells and not in B cells. Collectively, our findings suggest that genetic risk haplotypes need to be understood in the context of cell lineage-specific functionality. Thus, somewhat unexpectedly, in the case of B cells and based on our assays cell intrinsic effects of IRF5 haplotypes do not appear to be an important factor in risk for SLE. This, of course, does not rule out cell intrinsic effects that we have not studied and cell extrinsic effects of IRF5 haplotypes on B cell function, mediated perhaps through myeloid cell lineages (42).

We note that the literature provides some evidence that contrasts with our findings. In particular, the original definition of the IRF5 haplotypic diversity by Graham et al. showed that SLE risk alleles regulate expression of IRF5 in B lymphoblastoid cell lines. This was most clearly demonstrated for the rs10954213 allele that also regulates the formation of the short and long forms of the polyA tail of IRF5. Overall gene expression was assessed with Northern blot analysis or using microarray data in public databases, whereas our studies have utilized a TaqMan assay that is directed to the exon 2–3 boundary. Thus, it is possible that these two approaches may give different results in terms of overall quantitation of message due to differences in mRNA isoforms. Our approach to mRNA quantitation may have missed quantitative differences in the various mRNA splice variants that are encoded the risk and non-risk haplotypes. It is also possible that B cell lines have regulatory mechanisms that differ from native B cell populations. Nevertheless, our data strongly suggest that if mRNA splicing differences do exist between the risk and non-risk haplotypes, they do not substantially affect the B cell functional assays we have described here. Therefore, regardless of the mechanism, differences in haplotype transcriptional patterns do not appear to have a cell intrinsic effect on the critical B cell functions that we have investigated. It may well be that transcript isoforms do play some role in B cell functions that we have not examined, but our data address the major mechanisms that have been reported to be associated with B cell abnormalities in lupus.

These data emphasize the level of complexity that must be considered when trying to understand gene association studies with disease related phenotypes, namely that a gene may serve a critical purpose in a particular cell type, but a risk haplotype may or may not function differently from the non-risk haplotype in that cell type. The chain of causation in autoimmune diseases such as SLE clearly involves complex cell-cell interactions. The data reported here strongly suggest that the influence of IRF5 on B cell abnormalities is likely to depend to a large degree on the effects of IRF5 on cells which have a regulatory role on B cell function, such as myeloid cells. Further functional studies of IRF5 can profitably be focused on this possibility.

## Ethics Statement

The protocol was approved by the IRB of Northwell Health All subjects gave written informed consent in accordance with the Declaration of Helsinki.

## Author Contributions

JC: performed experiments, analyzed data, designed experiments, and wrote and edited manuscript. SR: built IRF5 haplotypes from 1000 Genomes data. PG: GAP and Biorepository leader, provided GAP samples and DNA for genotyping, and edited manuscript. BD: designed experiments, analyzed data, and edited manuscript.

## Conflict of Interest Statement

The authors declare that the research was conducted in the absence of any commercial or financial relationships that could be construed as a potential conflict of interest.

## Footnotes

^1^www.internationalgenome.org (Accessed: July, 2017).

^2^www.GAPRegistry.org (Accessed: August, 2013).

## References

[1] Sigurdsson S Nordmark G Goring HH Lindroos K Wiman AC Sturfelt G Polymorphisms in the tyrosine kinase 2 and interferon regulatory factor 5 genes are associated with systemic lupus erythematosus. Am J Hum Genet (2005) 76:528–37. 10.1086/428480 15657875 PMC1196404

[2] Graham RR Kozyrev SV Baechler EC Reddy MV Plenge RM Bauer JW A common haplotype of interferon regulatory factor 5 (IRF5) regulates splicing and expression and is associated with increased risk of systemic lupus erythematosus. Nat Genet (2006) 38:550–5. 10.1038/ng1782 16642019

[3] Krausgruber T Blazek K Smallie T Alzabin S Lockstone H Sahgal N IRF5 promotes inflammatory macrophage polarization and TH1-TH17 responses. Nat Immunol (2011) 12:231–8. 10.1038/ni.1990 21240265

[4] Mancl ME Hu G Sangster-Guity N Olshalsky SL Hoops K Fitzgerald-Bocarsly P Two discrete promoters regulate the alternatively spliced human interferon regulatory factor-5 isoforms. Multiple isoforms with distinct cell type-specific expression, localization, regulation, and function. J Biol Chem (2005) 280:21078–90. 10.1074/jbc.M500543200 15805103

[5] Krausgruber T Saliba D Ryzhakov G Lanfrancotti A Blazek K Udalova IA. IRF5 is required for late-phase TNF secretion by human dendritic cells. Blood (2010) 115:4421–30. 10.1182/blood-2010-01-263020 20237317

[6] Barnes BJ Moore PA Pitha PM. Virus-specific activation of a novel interferon regulatory factor, IRF-5, results in the induction of distinct interferon alpha genes. J Biol Chem (2001) 276:23382–90. 10.1074/jbc.M101216200 11303025

[7] Barnes BJ Kellum MJ Pinder KE Frisancho JA Pitha PM. Interferon regulatory factor 5, a novel mediator of cell cycle arrest and cell death. Cancer Res (2003) 63:6424–31. 14559832

[8] Hedl M Yan J Abraham C. IRF5 and IRF5 disease-risk variants increase glycolysis and human M1 macrophage polarization by regulating proximal signaling and Akt2 activation. Cell Rep (2016) 16:2442–55. 10.1016/j.celrep.2016.07.060 27545875 PMC5165654

[9] Kirou KA Lee C George S Louca K Papagiannis IG Peterson MG Coordinate overexpression of interferon-alpha-induced genes in systemic lupus erythematosus. Arthritis Rheum (2004) 50:3958–67. 10.1002/art.20798 15593221

[10] Feng X Wu H Grossman JM Hanvivadhanakul P FitzGerald JD Park GS Association of increased interferon-inducible gene expression with disease activity and lupus nephritis in patients with systemic lupus erythematosus. Arthritis Rheum (2006) 54:2951–62. 10.1002/art.22044 16947629

[11] Baechler EC Batliwalla FM Karypis G Gaffney PM Ortmann WA Espe KJ Interferon-inducible gene expression signature in peripheral blood cells of patients with severe lupus. Proc Natl Acad Sci U S A (2003) 100:2610–5. 10.1073/pnas.0337679100 12604793 PMC151388

[12] Bennett L Palucka AK Arce E Cantrell V Borvak J Banchereau J Interferon and granulopoiesis signatures in systemic lupus erythematosus blood. J Exp Med (2003) 197:711–23. 10.1084/jem.20021553 12642603 PMC2193846

[13] Feng D Stone RC Eloranta ML Sangster-Guity N Nordmark G Sigurdsson S Genetic variants and disease-associated factors contribute to enhanced interferon regulatory factor 5 expression in blood cells of patients with systemic lupus erythematosus. Arthritis Rheum (2010) 62:562–73. 10.1002/art.27223 20112383 PMC3213692

[14] Hedl M Abraham C. IRF5 risk polymorphisms contribute to interindividual variance in pattern recognition receptor-mediated cytokine secretion in human monocyte-derived cells. J Immunol (2012) 188:5348–56. 10.4049/jimmunol.1103319 22544929 PMC3409850

[15] Lien C Fang CM Huso D Livak F Lu R Pitha PM. Critical role of IRF-5 in regulation of B-cell differentiation. Proc Natl Acad Sci U S A (2010) 107:4664–8. 10.1073/pnas.0911193107 20176957 PMC2842054

[16] Yasuda K Watkins AA Kochar GS Wilson GE Laskow B Richez C Interferon regulatory factor-5 deficiency ameliorates disease severity in the MRL/lpr mouse model of lupus in the absence of a mutation in DOCK2. PLoS One (2014) 9:e103478. 10.1371/journal.pone.0103478 25076492 PMC4116215

[17] Yasuda K Nundel K Watkins AA Dhawan T Bonegio RG Ubellacker JM Phenotype and function of B cells and dendritic cells from interferon regulatory factor 5-deficient mice with and without a mutation in DOCK2. Int Immunol (2013) 25:295–306. 10.1093/intimm/dxs114 23291967 PMC3631000

[18] Savitsky DA Yanai H Tamura T Taniguchi T Honda K. Contribution of IRF5 in B cells to the development of murine SLE-like disease through its transcriptional control of the IgG2a locus. Proc Natl Acad Sci U S A (2010) 107:10154–9. 10.1073/pnas.1005599107 20479222 PMC2890425

[19] Tada Y Kondo S Aoki S Koarada S Inoue H Suematsu R Interferon regulatory factor 5 is critical for the development of lupus in MRL/lpr mice. Arthritis Rheum (2011) 63:738–48. 10.1002/art.30183 21305501

[20] Panchanathan R Liu H Liu H Fang CM Erickson LD Pitha PM Distinct regulation of murine lupus susceptibility genes by the IRF5/Blimp-1 axis. J Immunol (2012) 188:270–8. 10.4049/jimmunol.1102311 22116829 PMC3244553

[21] Avalos AM Uccellini MB Lenert P Viglianti GA Marshak-Rothstein A FcgammaRIIB regulation of BCR/TLR-dependent autoreactive B-cell responses. Eur J Immunol (2010) 40:2692–8. 10.1002/eji.200940184 20809520 PMC3060940

[22] Ravetch JV Bolland S. IgG Fc receptors. Annu Rev Immunol (2001) 19:275–90. 10.1146/annurev.immunol.19.1.275 11244038

[23] Bolland S Ravetch JV Inhibitory pathways triggered by ITIM-containing receptors. Adv Immunol (1999) 72:149–77. 10.1016/S0065-2776(08)60019-X 10361574

[24] Choubey D Panchanathan R. Interferon-inducible Ifi200-family genes in systemic lupus erythematosus. Immunol Lett (2008) 119:32–41. 10.1016/j.imlet.2008.06.001 18598717 PMC2585765

[25] Lazzari E Jefferies CA. IRF5-mediated signaling and implications for SLE. Clin Immunol (2014) 153:343–52. 10.1016/j.clim.2014.06.001 24928322

[26] Clark DN Read RD Mayhew V Petersen SC Argueta LB Stutz LA Four promoters of IRF5 respond distinctly to stimuli and are affected by autoimmune-risk polymorphisms. Front Immunol (2013) 4:360. 10.3389/fimmu.2013.00360 24223576 PMC3819785

[27] Alonso-Perez E Suarez-Gestal M Calaza M Kwan T Majewski J Gomez-Reino JJ Cis-regulation of IRF5 expression is unable to fully account for systemic lupus erythematosus association: analysis of multiple experiments with lymphoblastoid cell lines. Arthritis Res Ther (2011) 13:R80. 10.1186/ar3343 21627826 PMC3218890

[28] Clark DN Lambert JP Till RE Argueta LB Greenhalgh KE Henrie B Molecular effects of autoimmune-risk promoter polymorphisms on expression, exon choice, and translational efficiency of interferon regulatory factor 5. J Interferon Cytokine Res (2014) 34:354–65. 10.1089/jir.2012.0105 24350899

[29] Kozyrev SV Lewen S Reddy PM Pons-Estel B Argentine Collaborative Group Witte T Structural insertion/deletion variation in IRF5 is associated with a risk haplotype and defines the precise IRF5 isoforms expressed in systemic lupus erythematosus. Arthritis Rheum (2007) 56:1234–41. 10.1002/art.22497 17393452

[30] Graham RR Kyogoku C Sigurdsson S Vlasova IA Davies LR Baechler EC Three functional variants of IFN regulatory factor 5 (IRF5) define risk and protective haplotypes for human lupus. Proc Natl Acad Sci U S A (2007) 104:6758–63. 10.1073/pnas.0701266104 17412832 PMC1847749

[31] Cunninghame Graham DS Manku H Wagner S Reid J Timms K Gutin A Association of IRF5 in UK SLE families identifies a variant involved in polyadenylation. Hum Mol Genet (2007) 16:579–91. 10.1093/hmg/ddl469 17189288 PMC3706933

[32] Sigurdsson S Goring HH Kristjansdottir G Milani L Nordmark G Sandling JK Comprehensive evaluation of the genetic variants of interferon regulatory factor 5 (IRF5) reveals a novel 5 bp length polymorphism as strong risk factor for systemic lupus erythematosus. Hum Mol Genet (2008) 17:872–81. 10.1093/hmg/ddm359 18063667

[33] Guthridge JM Clark DN Templeton A Dominguez N Lu R Vidal GS Effects of IRF5 lupus risk haplotype on pathways predicted to influence B cell functions. J Biomed Biotechnol (2012) 2012:594056. 10.1155/2012/594056 22500098 PMC3304673

[34] Islam KB Baskin B Christensson B Hammarstrom L Smith CI. In vivo expression of human immunoglobulin germ-line mRNA in normal and in immunodeficient individuals. Clin Exp Immunol (1994) 95:3–9. 10.1111/j.1365-2249.1994.tb06006.x 8287606 PMC1534613

[35] Veri MC Gorlatov S Li H Burke S Johnson S Stavenhagen J Monoclonal antibodies capable of discriminating the human inhibitory Fcgamma-receptor IIB (CD32B) from the activating Fcgamma-receptor IIA (CD32A): biochemical, biological and functional characterization. Immunology (2007) 121:392–404. 10.1111/j.1365-2567.2007.02588.x 17386079 PMC2265948

[36] Genomes Project C Auton A Brooks LD Durbin RM Garrison EP Kang HM A global reference for human genetic variation. Nature (2015) 526:68–74. 10.1038/nature15393 26432245 PMC4750478

[37] Meffre E Wardemann H. B-cell tolerance checkpoints in health and autoimmunity. Curr Opin Immunol (2008) 20:632–8. 10.1016/j.coi.2008.09.001 18848883

[38] Hu G Mancl ME Barnes BJ. Signaling through IFN regulatory factor-5 sensitizes p53-deficient tumors to DNA damage-induced apoptosis and cell death. Cancer Res (2005) 65:7403–12. 10.1158/0008-5472.CAN-05-0583 16103093

[39] Farrant J Germ-line transcripts and class switching. Clin Exp Immunol (1994) 95:1–2. 10.1111/j.1365-2249.1994.tb06005.x 8287591 PMC1534646

[40] Mackay M Stanevsky A Wang T Aranow C Li M Koenig S Selective dysregulation of the FcgammaIIB receptor on memory B cells in SLE. J Exp Med (2006) 203:2157–64. 10.1084/jem.20051503 16923849 PMC2118390

[41] Deng Y Tsao BP. Advances in lupus genetics and epigenetics. Curr Opin Rheumatol (2014) 26:482–92. 10.1097/BOR.0000000000000086 25010439 PMC4222581

[42] Suurmond J Calise J Malkiel S Diamond B. DNA-reactive B cells in lupus. Curr Opin Immunol (2016) 43:1–7. 10.1016/j.coi.2016.07.002 27504587 PMC5125853

